# Taohong Siwu Decoction Ameliorates Ischemic Stroke Injury Via Suppressing Pyroptosis

**DOI:** 10.3389/fphar.2020.590453

**Published:** 2020-12-08

**Authors:** Mengmeng Wang, Zhuqing Liu, Shoushan Hu, Xianchun Duan, Yanyan Zhang, Can Peng, Daiyin Peng, Lan Han

**Affiliations:** ^1^ Anhui University of Chinese Medicine, Hefei, China; ^2^ Anhui Province Key Laboratory of Chinese Medicinal Formula, Hefei, China; ^3^ Anhui Medicine College, Hefei, China; ^4^ Key Laboratory of Xin’an Medicine, Ministry of Education, Hefei, China

**Keywords:** MAPK, HMGB1/toll-like receptors/NFκB, pyroptosis, ischemic stroke, Taohong Siwu decoction

## Abstract

**Objective:** Taohong Siwu decoction (THSWD) is one of the classic prescriptions for promoting blood circulation and removing blood stasis, and it has a good therapeutic effect on ischemic stroke. We sought to explore the therapeutic effects of THSWD on pyroptosis in rats with middle cerebral artery occlusion-reperfusion (MCAO/R).

**Methods:** MCAO/R model of rats were established by suture-occluded method. MCAO/R rats were randomly divided into five groups, which were model group, nimodipine group, THSWD high, medium and low dose group (18, 9, and 4.5 g/kg, respectively), rats of sham group without thread embolus. All rats were treated by intragastric administration for 7 days. We detected the level of inflammatory factors. NLRP3 and Caspase-1 were detected by immunofluorescence. Western blot was used to detect NLRP3, Caspase-1, ASC, and GSDMD in penumbra. Also, the expression of TXNIP, HMGB1, toll-like receptors (TLR4), NF-κB, and MAPK were detected.

**Results:** THSWD treatment improved the behavioral function and brain pathological damage. These results showed that the levels of TNF-α, TGF-β, IL-2, IL-6, IL-1β, and IL-18 were significantly reduced in THSWD treatment groups. THSWD could significantly decrease the expression levels of NLRP3, Caspase-1, Caspase-1 p10, ASC, TXNIP, GSDMD, HMGB1, TLR4/NFκB, p38 MAPK, and JNK in penumbra.

**Conclusion:** Our results showed that THSWD could reduce the activation level of NLRP3 inflammatory corpuscle, down-regulate GSDMD, and inhibit pyroptosis in MCAO/R rats. These may be affected by inhibiting HMGB1/TLR4/NFκB, MAPK signaling pathways.

## Introduction

In 2016, The Lancet reported that about 90.7% of strokes were related to 10 risk factors, including hypertension, smoking, dyslipidemia, alcohol intake, unhealthy diet, etc (O’Donnell et al., 2016). Although the mortality rate of stroke has shown a downward trend in China (Wang et al., 2017). However, stroke was an important cause of death and disability at present. The prevalence rate continues to increased, and the affected population has shown a younger trend (Fu et al., 2020). Therefore, the prevention and treatment of stroke was a very serious problem in China.

Mitochondrial dysfunction triggers cascade reactions after ischemia stroke, such as the generation of large amounts of endogenous ROS, inflammation, and autophagy. Inflammatory corpuscle and pyroptosis are important in stroke (Fann et al., 2013; Barrington et al., 2017; Dong et al., 2018). The activated NF-κB pathway up-regulates the gene expression of NLRP3, pro-IL-1β, and pro-IL-18 (Bauernfeind et al., 2009). The production of endogenous ROS and cathepsin B could stimulate self-assembly of NLRP3 inflammatory corpuscle (Chen and Sun, 2013; Latz et al., 2013). NLRP3, ASC, and pro-Caspase-1 have been assembled to form a protein complex, which promoted cleavage to produce activated Caspase-1. GSDMD is cleaved by mature Caspase-1. GSDMD N-terminal domain assembles membrane pores to induce pyroptosis (Shi et al., 2015; Mulvihill et al., 2018). Mature IL-1β and IL-18 are released extracellularly through GSDMD membrane pores (He et al., 2015). At the same time, the contents such as HMGB1 are released to the outside of the cell. HMGB1 binds to the transmembrane receptors RAGE and toll-like receptors (TLR4), and further activates the nuclear transcription factor NF-κB, which further enhances the inflammatory response (Paudel et al., 2019).

Ischemic stroke is considered to be the cerebrovascular disease (Rutten-Jacobs and Rost, 2019). Traditional Chinese medicine has been widely used to treat vascular diseases for nearly 2000 years. Taohong Siwu decoction (THSWD) originated from the traditional Chinese medicine book *Yizong Jinjian* of the Qing Dynasty, which is composed of *Prunus persica* (L.) Batsch, *Carthamus tinctorius* L, *Angelica sinensis* (Oliv.) Diels, *Rehmannia glutinosa* (Gaertn.) DC., *Ligusticum chuanxiong* Hort, *Paeonia lactiflora* Pall. (Table 1). THSWD has been widely used clinically to treat vascular diseases in the past. Our research showed that THSWD could reduce oxidative stress injury, improve learning and memory function, and promote angiogenesis in ischemic stroke (Han et al., 2015; Chen et al., 2020; Wang M. et al., 2020). It is well known that inflammation and pyroptosis are involved in ischemic stroke injury (Barrington et al., 2017; Dong et al., 2018). There is no reported about the effect of THSWD on pyroptosis. The mechanisms whereby THSWD is of value has not been fully elucidated in the treatment of ischemic stroke. Therefore, we hypothesized that THSWD could reduce pyroptosis in ischemic stroke.

**TABLE 1:** Contents of TaoHong SiWu decoction (THSWD).

Latin name	Chinese name	Part used	Weight (g)
*Prunus persica* (L.) Batsch	Tao Ren	Semen	9
*Carthamus tinctorius* L.	Hong Hua	Flos	6
*Angelica sinensis* (Oliv.) Diels	Dang Gui	Radix	9
*Rehmannia glutinosa* (Gaertn.) DC.	Shu Di Huang	Radix	12
*Ligusticum chuanxiong* Hort.	Chuan Xiong	Rhizoma	6
*Paeonia lactiflora* Pall.	Bai Shao	Radix	9

In present study, we studied the effect of THSWD on inflammatory factors in rats with middle cerebral artery occlusion-reperfusion (MCAO/R). More importantly, we further explored the effect of THSWD on pyroptosis. This is more conducive to the research and development of THSWD as a candidate drug for the treatment of stroke.

## Materials and Methods

### Materials

Tao Ren (*Prunus persica* (L.) Batsch, 1702181), Hong Hua (*Carthamus tinctorius* L., 17072135.), Dang Gui (*Angelica sinensis* (Oliv.) Diels, 1611085), Shu Di Huang (*Rehmannia glutinosa* (Gaertn.) DC., 1705312), Chuan Xiong (*Ligusticum chuanxiong* Hort, 17010335), Bai Shao (*Paeonia lactiflora* Pall., 17110114) were purchased from Bozhou Yonggang Pieces Factory Co., Ltd. (Bozhou, China). They were verified by Qingshan Yang (Anhui University of Chinese Medicine, Hefei, China). Nimodipine (State Food and Drug Administration approval number: H14022821) was purchased from Yabao Pharmaceutical Group CO., Ltd. (Yuncheng, China).

Anti-body (GSDMD:ab219800, Caspase-1:ab1872, Caspase-1 p10:ab179515, HMGB1:ab77302, JNK:ab124956, p38:ab45381, TLR4:ab217274) were purchased from Abcam (Cambridge, MA, United States). Anti-body (ASC:sc-514414, TXNIP:sc-271238) were bought from Santa Cruz Biotechnology (Santa Cruz, CA, United States). Anti-NLRP3 (NBP2-12446) was bought from Novus (Colorado, United States). Anti-NF-κB (bs-0465R) was bought from Bioss (Beijing, China). Anti-GAPDH (19AF0406) was purchased from ZSGB-BIO (Beijing, China). Rat TNF-α, IL-6, IL-1β, and IL-18 Elisa kits (201903) were purchased from Meimian (Jiangsu, China), rat IL-2, and TGF-β Elisa kits (GR2019-03) were purchased from JYM (Wuhan China).

### Preparation of Taohong Siwu Decoction

These THSWD were mixed of six herbs in the proportion of Table 1, soaked in 10 times (v/w) 75% ethanol for 2 h, boiled and refluxed for 2 h. Then the filtrate was collected, and the residue was refluxed with 8 times the amount of 75% ethanol for 2 h. The two filtrates were mixed and concentrate to 1.8 g/ml by rotary evaporation. According to the published standard experimental procedure, UPLC was used to ensure the quality and stability of the THSWD (Han et al., 2017; Chen et al., 2020). The assay chromatogram of THSWD of the same batch number and preparation has been published (Chen et al., 2020).

### Animals and Middle Cerebral Artery Occlusion Surgery

Healthy male Sprague–Dawley rats weighing 270 ± 20 g were provided by the Experimental Animal Center of Anhui Medical University (Hefei, China). All rats were housed in a polypropylene cage (25 ± 5°C, 50–60% relative humidity) under controlled lighting (12 h light/dark cycle), and allowed free access to food and water.

All experimental rats were anesthetized by pentobarbital (50 mg/kg, i.p.). The common carotid artery (CCA), external carotid artery (ECA), and internal carotid artery (ICA) were carefully separated from the middle cervical incision of the rat neck. To ensure the middle cerebral artery (MCA) was occluded, an incision was made in the CCA, and the nylon suture was inserted to about 18–20 mm through the ICA. The nylon suture was a polylysine coated monofilament nylon with a diameter of 0.285 mm. After 2 h of surgery, the nylon suture was withdrawn from MCA and reperfused. After 2 h of surgery, the nylon suture was withdrawn to allow reperfused. The sham rats only performed the same process of separating blood vessels. The temperature was kept at 37°C in the experimental process.

After 24 h of ischemia-reperfusion, the behavioral scores were performed according to the method of Zea Longa, and rats were randomly divided into six groups: Sham, MCAO, THSWD (18, 9 and 4.5 g/kg, respectively, equivalent to the dry weight of the raw materials), nimodipine (20 mg/kg) groups, and treated (i.g.) for 7 days.

### Functional Outcome Assessment

On the 7th day after the rats were given treatment, all rats completed the Bederson scores. 0, no observable deficit. 1, forelimb flexion when suspended by the tail. 2, decreased resistance to push. 3, counterclockwise circling. 4, unconsciousness, including death within 24 h.

### Measurement of Infarct Volume

After killing the rats, brains were isolated and sectioned into five coronal slices in 2 mm thickness. Which were stained with 2,3,5-Triphenyltetrazolium chloride (TTC, 2%, T8170, Solarbio, Beijing, China) for 30 min under dark conditions of 37°C. The coronal slices were taken pictures through digital camera and analyze the infarct volume by Image J (NIH, Bethesda, MD, United States).

### Histomorphological Analysis

The brains were fixed in 4% paraformaldehyde, and paraffin sections were prepared for HE staining (BA-4041, BA-4024, BASO, Zhuhai China). After sealed with neutral resin, and images were captured using an optical microscope (SYZX6061, Nikon, Tokyo, Japan). The histomorphological analysis was evaluated by two examiners blinded to the treatment groups.

### ELISA

Rat penumbra tissues were separated and homogenized with 10 times PBS (v/w) on ice. The homogenates were centrifuged at 4,000 rpm for 10 min at 4°C, and supernatants were collected, then stored in −80°C until future use. The secretion levels of inflammatory cytokines (TNF-α, IL-2, IL-6, TGF-β, IL-1β, IL-18) were analyzed by ELISA. According to the manufacturer’s protocol above in the ELISA kit instructions, the optical density (OD) at 450 nm was measured by enzyme-labeled instrument (Multiskan GO, Thermo, Waltham, MA, United States).

### Immunofluorescence Staining

The brain tissues were quickly removed and frozen, and frozen sections were made. The sections were reacted with a primary antibody and subsequently reacted with a fluorescently labeled secondary antibody. Then, which were sealed with a sealer containing a quencher. Two examiners blinded to the treatment groups were observed and photographed using a fluorescence microscope (ECLIPSE TI-SR, Nikon, Tokyo, Japan), and analyzed by ImageJ.

### Western Blotting

Rat penumbra tissues were separated and homogenized with 10 times ice-cold lysis buffer containing protein inhibitor. The homogenates were centrifuged at 12,000 rpm for 10 min at 4°C, and supernatants were collected. The protein concentration of supernatants were measured by BCA kit (PICPI23223, Thermo, Waltham, MA, United States). In order to denature the protein, the protein samples were added to buffer and boiled for 10 min at 100°C. Equal amounts of proteins were separated by electrophoresis, transferred to NC membranes at low temperatures. After blocking with 5% skimmed milk powder for 2 h, membranes were incubated overnight at 4°C with primary antibodies (anti-NLRP3, anti-ASC, anti-Caspase-1, anti-TXNIP, anti-Caspase-1 p10, anti-GAPDH). The NC membranes were then incubated with the secondary antibody (HRP-conjugated anti-rabbit and anti-mouse secondary antibody, A21010, Abbkine, Wuhan China), and developed with enhanced chemiluminescence (ECL, 32109, Thermo, Waltham, MA, United States).

### Statistical Analysis

The data were analyzed with SPSS 23.0 software and expressed as the mean ± SD. Statistical analyses were performed using one-way ANOVA, followed by LSD tests for the significance of the difference between groups. *p <* 0.05 was considered statistically significant.

## Results

### Effects of Taohong Siwu Decoction on the Neurological Defect Scores and Infarction Volume in Middle Cerebral Artery Occlusion-Reperfusion Model

**FIGURE 1:**
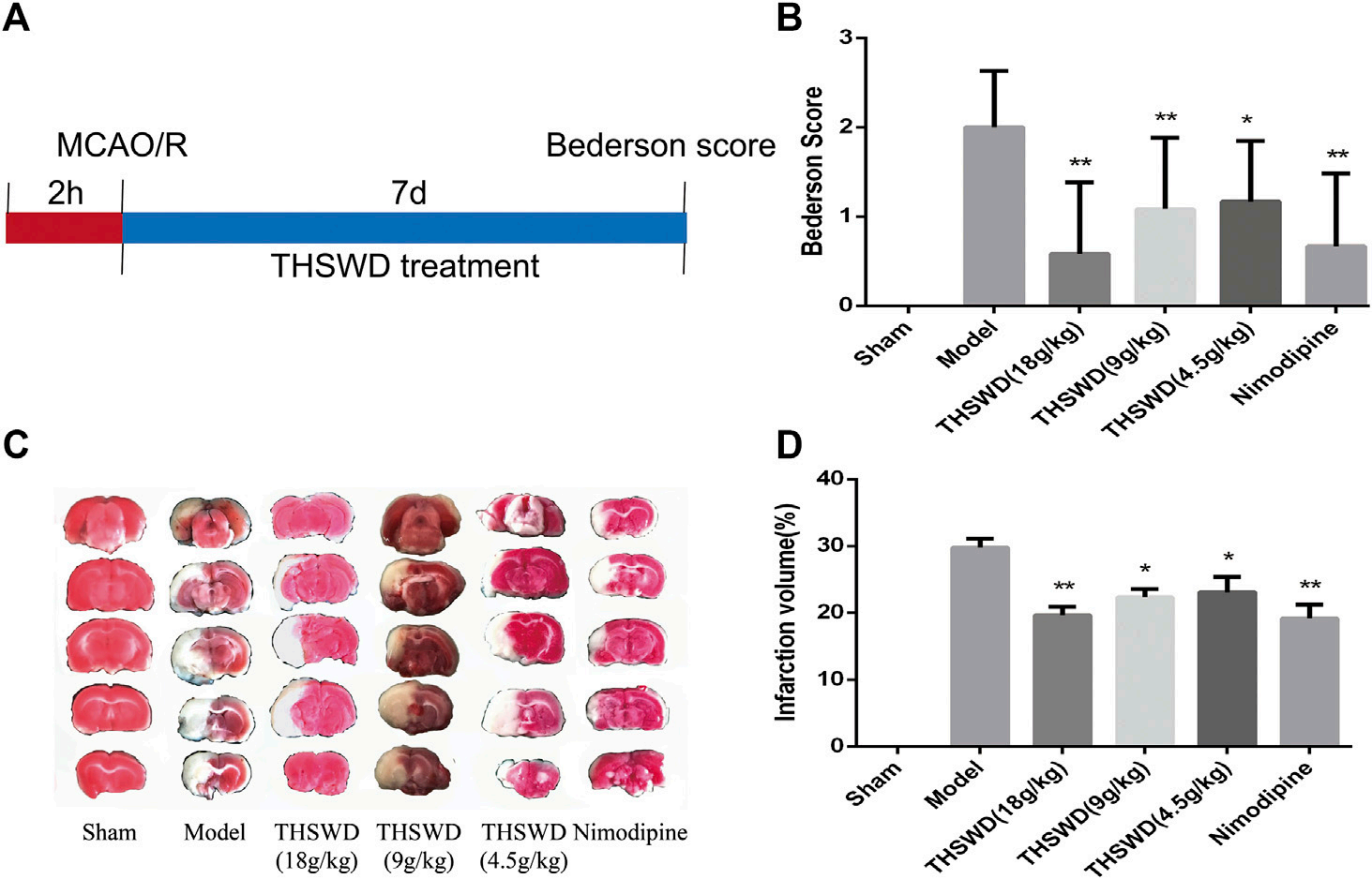
Taohong Siwu decoction (THSWD) alleviated middle cerebral artery occlusion-reperfusion (MCAO/R) induced brain damage. **(A)** The flow diagram of the experiment. **(B)** Neurological deficits scores. **(C)** 2,3,5-Triphenyltetrazolium chloride staining of representative sections. **(D)** Quantification of infarction volume rates. The results were presented as the mean ± SD (*n* = 6). Compared with sham group, ^#^ *p* < 0.05, ^##^ *p* < 0.01. Compared with model group, *p < 0.05, **p < 0.01.

As shown in Figure 1B, after 7 days of treatment with THSWD and nimodipine, the behavioral function were significantly improved of MCAO/R rats. Compared with model group, the Berderson scores of THSWD and nimodipine groups were significantly reduced (*p <* 0.05, *p <* 0.01). Sham group rats had no behavioral function impairment. TTC staining was used to calculate the infarct volume of rats. The brain of sham group rats had no infarction volume. Compared with model group, the infarct volume were significantly reduced of THSWD and the nimodipine treatment group (Figures 1C,D).

### Effect of Taohong Siwu Decoction on the Level of Pathological Damage in Brain of Middle Cerebral Artery Occlusion-Reperfusion Rats

**FIGURE 2:**
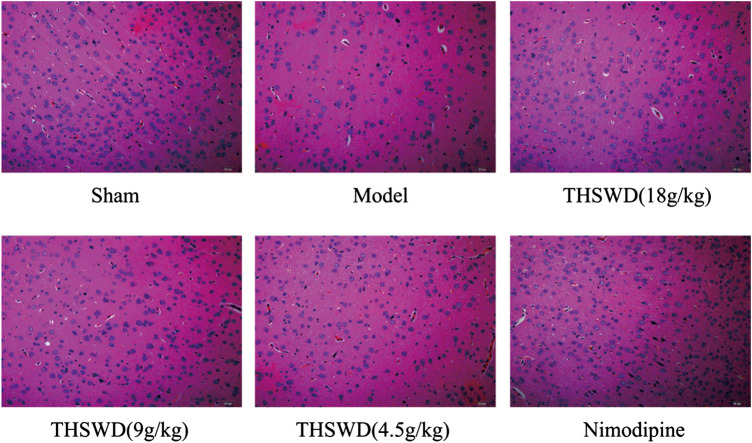
Effect of Taohong Siwu decoction (THSWD) on the level of pathological damage in middle cerebral artery occlusion-reperfusion (MCAO/R) rats (×200, *n* = 3).

The pathological damage of brain tissue was observed by HE staining. The specific results showed in Figure 2. The positive cells were intact and abundant, and no infiltration of inflammatory cells in sham group. The model group showed that the number of positive cells nuclei were significantly reduced, most cells exhibited visible disorder. There were phenomena, such as nuclear shrinkage, nuclear rupture, and inflammatory cells infiltrated. Advantageously, THSWD treatment group significantly alleviated the abnormal phenomena caused by MCAO/R.

### Effect of Taohong Siwu Decoction on Inflammatory Factors in Middle Cerebral Artery Occlusion-Reperfusion Rats

**FIGURE 3:**
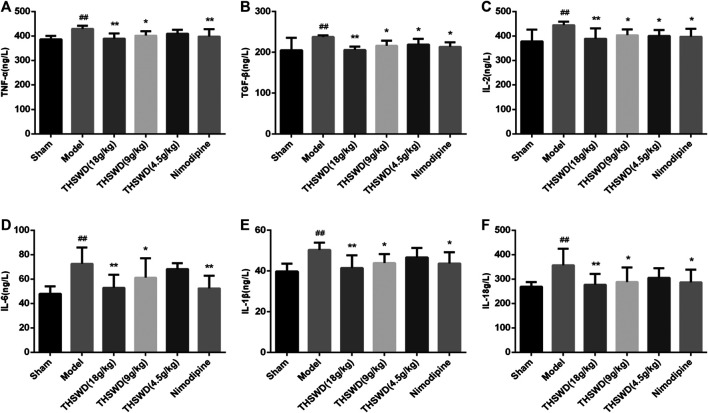
Taohong Siwu decoction (THSWD) reduced the levels of inflammatory factors in middle cerebral artery occlusion-reperfusion (MCAO/R) rats. **(A)** TNF-α, **(B)** TGF-β, **(C)** IL-2, **(D)** IL-6, **(E)** IL-1β, **(F)** IL-18. The results were presented as the mean ± SD (*n* = 6). Compared with sham group, ^#^ *p* < 0.05, ^##^ *p* < 0.01. Compared with model group, *p < 0.05, **p < 0.01.

Pro-inflammatory factors increased after a stroke. Besides, plenty of inflammatory factors were detrimental to the function of tissues and cells. We detected the levels of inflammatory factors in penumbra by ELISA. Compared with sham group, the levels of TNF-α, IL-2, IL-6, TGF-β, IL-1β, and IL-18 were significantly increased in model group (*p <* 0.05, *p <* 0.01). Compared with model group, THSWD and nimodipine treatment groups significantly reduced the level of inflammatory factors (*p <* 0.05, *p <* 0.01). These showed that THSWD could attenuate inflammatory response of MCAO/R rats (Figure 3). During the occurrence of pyroptosis, IL-1β and IL-18 were secreted to enhance the inflammatory response. Therefore, we further investigated the effect of THSWD on pyroptosis in MCAO/R rats.

### Effect of Taohong Siwu Decoction on Pyroptosis in Middle Cerebral Artery Occlusion-Reperfusion Rats

**FIGURE 4:**
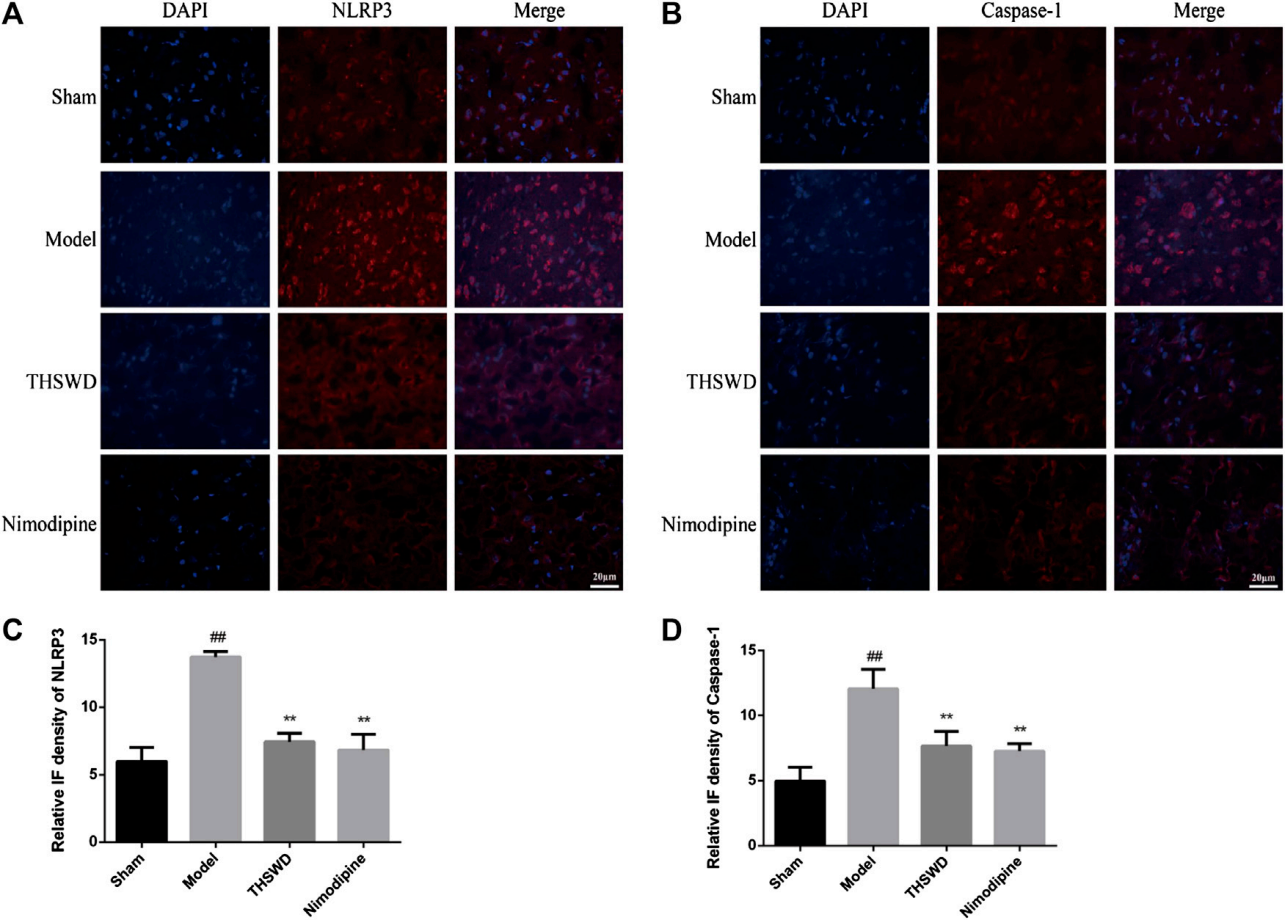
Taohong Siwu decoction (THSWD) inhibited NLRP3 and Caspase-1 expression (×400). **(A)** NLRP3, **(B)** Caspase-1. The nuclei were stained blue by DAPI, and NLRP3 and Caspase-1 were red. **(C,D)** Fluorescence intensity analysis of NLRP3 and Caspase-1 staining (*n* = 3).

Immunofluorescence was used to detect the expression of NLRP3 and Caspase-1 in brain. We observed the picture qualitatively and draw the following preliminary results. As shown in Figure 4, compared with sham group, the fluorescence intensity of NLRP3 and Caspase-1 increased significantly in model group. Compared with model group, the fluorescence intensity of NLRP3 and Caspase-1 decreased in THSWD (18 g/kg) treatment groups. We also advanced quantitative analysis of NLRP3 and Caspase-1 by western blot.

**FIGURE 5:**
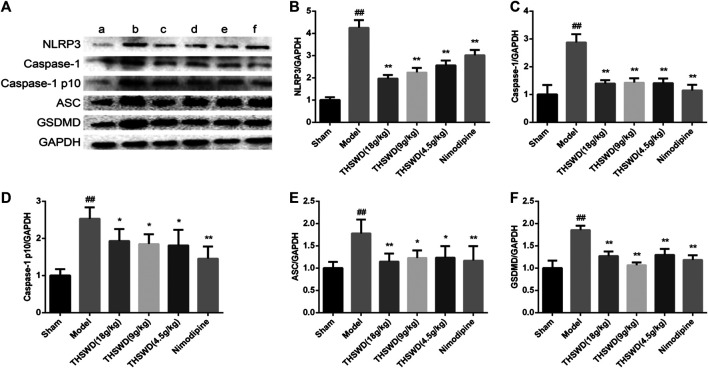
Effect of Taohong Siwu decoction (THSWD) on the characteristic protein of pyroptosis in middle cerebral artery occlusion-reperfusion (MCAO/R) rats. **(A)** Photographs of western blots, **(B)** NLRP3, **(C)** Caspase-1, **(D)** Caspase-1 p10, **(E)** ASC, **(F)** GSDMD. **a**: Sham, **b**: Model, **c**: THSWD (18 g/kg), **d**: THSWD (9 g/kg), **e**: THSWD (4.5 g/kg), **f**: nimodipine. The results were presented as the mean ± SD (*n* = 3). Compared with sham group, ^#^ *p* < 0.05, ^##^ *p* < 0.01. Compared with model group, *p < 0.05, **p < 0.01.

We detected the expression levels of NLRP3 inflammatory corpuscle constituent protein and pyroptosis executive protein in penumbra. As shown in Figure 5, these results showed that compared with sham group, the levels of NLRP3, Caspase-1, Caspase-1 p10, ASC, and GSDMD were significantly increased (*p <* 0.01) in model group. Compared with model group, the levels of NLRP3, Caspase-1, Caspase-1 p10, ASC, and GSDMD were significantly reduced (*p <* 0.05, *p <* 0.01) of THSWD and nimodipine treatment groups in penumbra. Our results indicated that THSWD could inhibit the activation of NLRP3 inflammatory corpuscle and inhibit pyroptosis in MCAO/R rats.

### Taohong Siwu Decoction Inhibited the Activity of HMGB1-Toll-Like Receptors-NFκB and MAPK Signaling Pathways

**FIGURE 6:**
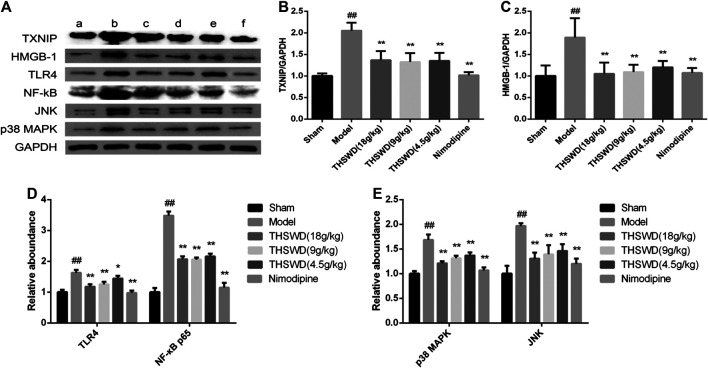
Effect of Taohong Siwu decoction (THSWD) on the pathways of regulation of pyroptosis in middle cerebral artery occlusion-reperfusion (MCAO/R) rats. **(A)** Photographs of western blots, **(B)** TXNIP, **(C)** HMGB-1, **(D)** toll-like receptors-NFκB, **(E)** MAPK. **a:** Sham, **b:** Model, **c:** THSWD (18 g/kg), **d:** THSWD (9 g/kg), **e:** THSWD (4.5 g/kg), **f:** nimodipine. The results were presented as the mean ± SD (*n* = 3). Compared with sham group, ^#^ *p* < 0.05, ^##^ *p* < 0.01. Compared with model group, *p < 0.05, **p < 0.01.

To explore how THSWD inhibits pyroptosis, western blot was used to detect the signaling pathway in penumbra of the brain. These results showed that compared with sham group, the levels of TXNIP, HMGB1, TLR4 and NF-κB p65 were significantly increased (*p <* 0.01) in model group. Compared with model group, the levels of TXNIP, HMGB1, TLR4, and NF-κB p65 were significantly reduced (*p <* 0.05, *p <* 0.01) in THSWD and nimodipine treatment groups (Figures 6B–D). This indicated that THSWD inhibited pyroptosis through down-regulating the HMGB1-TLR4-NFκB pathway.

In order to study the effect of THSWD on the MAPK pathway of MCAO/R rats, the expression of JNK and p38 MAPK were detected in penumbra. These results showed compared with sham group, the levels of JNK and p38 MAPK were significantly increased (*p <* 0.01) in MCAO/R rats. Compared with model group, the levels of JNK and p38 MAPK were significantly reduced (*p <* 0.05, *p <* 0.01) in THSWD and nimodipine treatment groups (Figure 6E). This indicated that THSWD inhibited pyroptosis through down-regulating the MAPK pathway.

## Discussion

The inflammatory response is activated due to vascular occlusion in ischemic stroke. Leukocytes are recruited into endothelial cells, which damages the blood-brain barrier (BBB) and a large number of inflammatory mediators are released (Anrather and Iadecola, 2016; De Meyer et al., 2016). Intravascular inflammation is the basis of BBB breakdown and leukocyte invasion. At the same time, the inflammatory cascade process started in brain parenchyma. Microglia are recruited near damaged blood vessels and they are activated quickly (Szalay et al., 2016). Inflammatory factors are released such as IL-1β and TNF-α and fed back into the inflammatory cascade through other immune cells (Anrather and Iadecola, 2016; Szalay et al., 2016). Our previous research has proved that THSWD has a good therapeutic effect on MCAO/R. This study also confirms previous conclusions. In this study, the results showed that THSWD could reduce the levels of inflammatory factors in MCAO/R rats. This provides a positive signal for exploring the role of THSWD in pyroptosis.

Pyroptosis is widely involved in central nervous system diseases. Unlike apoptosis, pyroptosis occurred faster and accompanied by the release of a large number of inflammatory factors. Many studies have shown that almost all N-terminal domains of Gasdermin family proteins could induce pyroptosis. GSDMD is the common substrate for activated Caspase-1 and Caspase-4/5/11 (Wang K. et al., 2020). The domain of GSDMD between N-terminal domain and C-terminal domain is cleaved by activated Caspase. GSDMD N-terminal domain specifically is bound to cardiolipin and phosphoinositide, recruited oligomerization on the plasma membrane to form a membrane pore. GSDMD C-terminal domain could inhibit GSDMD N-terminal domain to maintain GSDMD inhibition state (Liu et al., 2018). NLRP3 inflammatory corpuscle is the most widely studied all inflammatory corpuscles. A large number of studies have shown that NLRP3 is expressed among neurons, endothelial cells, and microglia. TXNIP is an endogenous inhibitor of TRX. After cells are stimulated by inflammatory corpuscle activators (ROS), the oxidized TRX causes TXNIP/TRX decomposition. TXNIP is shuttled to cytoplasmic mitochondria in the ROS-dependent manner, which bound to NLRP3, and activates NLRP3 inflammatory corpuscle (Zhou et al., 2011; Nasoohi et al., 2018). Our direct observation of immunofluorescence results showed that compared with sham group, the expression levels of NLRP3 and Caspase-1 increased in MCAO/R group. Compared with MCAO/R group, the expression of NLRP3 and Caspase-1 decreased in the THSWD (18 g/kg) treatment group. Further research confirmed that THSWD could significantly decrease the expression levels of NLRP3, Caspase-1, GSDMD, TXNIP, ASC. These results proved that pyroptosis is activated by MCAO/R. THSWD could reduce the activation of NLRP3 inflammatory corpuscle and inhibit pyroptosis.

HMGB1 is transferred from the nucleus to the cytoplasm, which is secreted extracellularly by activating inflammatory corpuscles (Vande Walle et al., 2011). Outside the cell, HMGB1 bound to its receptor (TLR2, TLR4, TLR9, RAGE), mediated the production of downstream inflammatory factors and expanded the inflammatory response. Studies have shown that HMGB1 could induce the formation of NLRP3 inflammatory corpuscle through TLRs (Song et al., 2017; Yu et al., 2019). The pro-inflammatory HMGB1-TLR4-NLRP3-GSDMD signal axis could induce Caspase-1 mediated pyroptosis (Dong et al., 2019). Signaling molecules downstream of TLR4/MyD88 pathway include NF-κB, JNK, p38 MAPK, and ERK1/2. JNK and p38 MAPK are mainly activated by various cellular stress signals and pro-inflammatory cytokines (Kim and Choi, 2015). In ischemic stroke, MAPK and NF-κB signaling pathways are key links in the expression and activation of NLRP1 and NLRP3 inflammatory corpuscles (Fann et al., 2018). Their involvement is widely recognized in activating inflammatory corpuscles. In this study, we have demonstrated that HMGB1/TLR4/NFκB and MAPK were activated in the MCAO/R rats. THSWD could inhibit the activation of HMGB1/TLR4/NFκB and MAPK.

Our team conducted the THSWD fingerprint study by UPLC. A total of fifteen compounds were identified. The six compounds were initially compared by standard product, including hydroxysafflor yellow a, 5-hydroxymethyl furfuraldehyde, ferulic acid, ligustilide, amygdalin and paeoniflorin (Han et al., 2017). The assay chromatogram of THSWD of the same batch number and preparation has been published (Chen et al., 2020). Their respective contents of hydroxysafflor yellow A, amygdalin, paeoniflorin, ferulic acid, verbascoside, and ligustilide in THSWD were identified as 0.198, 0.45, 0.602, 0.031, 0.014, and 0.256 mg ml^−1^ (Chen et al., 2020). Also, our previous report has investigated the major constituents of THSWD by UPLC-Q-TOF-MS. A total of 95 components have been identified, including aromatic acids, flavonoids, polysaccharides, volatile oils, monoterpene glycosides, aromatic cyanoglycosides (Duan et al., 2019). Many published articles have confirmed that they are the basis of THSWD inhibitors of pyroptosis (Liu et al., 2017; Ye et al., 2020; Yin et al., 2020).

In summary, the findings showed that THSWD could significantly reduce the level of inflammatory factors. Additionally, this study demonstrated that pyroptosis is involved in MCAO/R rats. THSWD exerts significant effects on ischemic brain injury through a mechanism closely related to reduce the activation of NLRP3 inflammatory corpuscle and inhibit pyroptosis. These may be achieved by down-regulating the HMGB1-TLR4-NFκB and MAPK pathways. This study is of great significance to verify the main efficacy of traditional Chinese medicine THSWD, as it confirms the mechanism of action of the THSWD on pyroptosis. This work is conducive to the research and development of the stroke candidate drugs of THSWD.

## Data Availability Statement

The raw data supporting the conclusions of this article will be made available by the authors, without undue reservation.

## Ethics Statement

The principles of laboratory animal care followed the guiding principles for the care and use of laboratory animals. All experimental procedures were authorized by the Committee on the Ethics of Animal Experiments of Anhui University of Chinese Medicine.

## Author Contributions

MW, CP, DP, and LH designed and supervised the study. MW and ZL performed the experiments. SH, XD, and YZ analyzed the data. MW and ZL wrote the paper.

## Funding

This work was supported by grants from the National Natural Science Foundation of China (Nos. 82074152, 81903953, 81503291, and 81473387).

## Conflict of Interest

The authors declare that the research was conducted in the absence of any commercial or financial relationships that could be construed as a potential conflict of interest.

## References

[B1] Anrather J. Iadecola C. (2016). Inflammation and stroke: an overview. Neurotherapeutics 13 (4), 661–670. 10.1007/s13311-016-0483-x 27730544 PMC5081118

[B2] Barrington J. Lemarchand E. Allan S. M. (2017). A brain in flame; do inflammasomes and pyroptosis influence stroke pathology? Brain Pathol. 27 (2), 205–212. 10.1111/bpa.12476 27997059 PMC8028888

[B3] Bauernfeind F. G. Horvath G. Stutz A. Alnemri E. S. Macdonald K. Speert D. (2009). Cutting edge: NF-κB activating pattern recognition and cytokine receptors license NLRP3 inflammasome activation by regulating NLRP3 expression. J. Immunol. 183 (2), 787–791. 10.4049/jimmunol.0901363 19570822 PMC2824855

[B4] Chen F.-F. Wang M.-M. Xia W.-W. Peng D.-Y. Han L. (2020). Tao-Hong-Si-Wu decoction promotes angiogenesis after cerebral ischaemia in rats via platelet microparticles. Chin. J. Nat. Med. 18 (8), 620–627. 10.1016/s1875-5364(20)30074-1 32768169

[B5] Chen S. Sun B. (2013). Negative regulation of NLRP3 inflammasome signaling. Protein Cell 4 (4), 251–258. 10.1007/s13238-013-2128-8 23519777 PMC4875520

[B6] De Meyer S. F. Denorme F. Langhauser F. Geuss E. Fluri F. Kleinschnitz C. (2016). Thromboinflammation in stroke brain damage. Stroke 47 (4), 1165–1172. 10.1161/strokeaha.115.011238 26786115

[B7] Dong W. Zhu Q. Yang B. Qin Q. Wang Y. Xia X. (2019). Polychlorinated biphenyl quinone induces caspase 1-mediated pyroptosis through induction of pro-inflammatory HMGB1-TLR4-NLRP3-GSDMD signal axis. Chem. Res. Toxicol. 32 (6), 1051–1057. 10.1021/acs.chemrestox.8b00376 30977640

[B8] Dong Z. Pan K. Pan J. Peng Q. Wang Y. (2018). The possibility and molecular mechanisms of cell pyroptosis after cerebral ischemia. Neurosci. Bull. 34 (6), 1131–1136. 10.1007/s12264-018-0294-7 30306532 PMC6246843

[B9] Duan X. Pan L. Bao Q. Peng D. (2019). UPLC-Q-TOF-MS study of the mechanism of THSWD for breast cancer treatment. Front. Pharmacol. 10, 1625 10.3389/fphar.2019.01625 32038266 PMC6993183

[B10] Fann D. Y. Lee S. Y. Manzanero S. Tang S. C. Gelderblom M. Chunduri P. (2013). Intravenous immunoglobulin suppresses NLRP1 and NLRP3 inflammasome-mediated neuronal death in ischemic stroke. Cell Death Dis. 4, e790 10.1038/cddis.2013.326 24008734 PMC3789184

[B11] Fann D. Y.-W. Lim Y.-A. Cheng Y.-L. Lok K.-Z. Chunduri P. Baik S.-H. (2018). Evidence that NF-κB and MAPK signaling promotes NLRP inflammasome activation in neurons following ischemic stroke. Mol. Neurobiol. 55 (2), 1082–1096. 10.1007/s12035-017-0394-9 28092085

[B12] Fu H. Zhang D. Zhu R. Cui L. Qiu L. Lin S. (2020). Association between lipoprotein(a) concentration and the risk of stroke in the Chinese Han population: a retrospective case-control study. Ann. Transl. Med. 8 (5), 212 10.21037/atm.2020.01.38 32309359 PMC7154407

[B13] Han L. Ji Z. Chen W. Yin D. Xu F. Li S. (2015). Protective effects of tao-Hong-si-Wu decoction on memory impairment and hippocampal damage in animal model of vascular dementia. Evid Based Compl. Alternat. Med. 2015, 195835 10.1155/2015/195835 PMC4363643 25821478

[B14] Han L. Qiao O. Wu H. Wu S. Zhang Y. Yao L. (2017). Chromatographic fingerprint analysis is feasible for comprehensive quality control of Taohongsiwu. Int. J. Pharmacol. 13 (5), 488–494. 10.3923/ijp.2017.488.494

[B15] He W.-T. Wan H. Hu L. Chen P. Wang X. Huang Z. (2015). Gasdermin D is an executor of pyroptosis and required for interleukin-1β secretion. Cell Res. 25 (12), 1285–1298. 10.1038/cr.2015.139 26611636 PMC4670995

[B16] Kim E. K. Choi E.-J. (2015). Compromised MAPK signaling in human diseases: an update. Arch. Toxicol. 89 (6), 867–882. 10.1007/s00204-015-1472-2 25690731

[B17] Latz E. Xiao T. S. Stutz A. (2013). Activation and regulation of the inflammasomes. Nat. Rev. Immunol. 13 (6), 397–411. 10.1038/nri3452 23702978 PMC3807999

[B18] Liu Y.-M. Shen J.-D. Xu L.-P. Li H.-B. Li Y.-C. Yi L.-T. (2017). Ferulic acid inhibits neuro-inflammation in mice exposed to chronic unpredictable mild stress. Int. Immunopharm. 45, 128–134. 10.1016/j.intimp.2017.02.007 28213267

[B19] Liu Z. Wang C. Rathkey J. K. Yang J. Dubyak G. R. Abbott D. W. (2018). Structures of the gasdermin D C-terminal domains reveal mechanisms of autoinhibition. Structure 26 (5), 778.e773–784.e773. 10.1016/j.str.2018.03.002 29576317 PMC5932255

[B20] Mulvihill E. Sborgi L. Mari S. A. Pfreundschuh M. Hiller S. Müller D. J. (2018). Mechanism of membrane pore formation by human gasdermin‐D. EMBO J. 37 (14), e98321 10.15252/embj.201798321 29898893 PMC6043855

[B21] Nasoohi S. Ismael S. Ishrat T. (2018). Thioredoxin-interacting protein (TXNIP) in cerebrovascular and neurodegenerative diseases: regulation and implication. Mol. Neurobiol. 55 (10), 7900–7920. 10.1007/s12035-018-0917-z 29488135 PMC6388721

[B22] O’donnell M. J. Chin S. L. Rangarajan S. Xavier D. Liu L. Zhang H. (2016). Global and regional effects of potentially modifiable risk factors associated with acute stroke in 32 countries (INTERSTROKE): a case-control study. Lancet 388 (10046), 761–775. 10.1016/s0140-6736(16)30506-2 27431356

[B23] Paudel Y. N. Angelopoulou E. Piperi C. Balasubramaniam V. R. M. T. Othman I. Shaikh M. F. (2019). Enlightening the role of high mobility group box 1 (HMGB1) in inflammation: updates on receptor signalling. Eur. J. Pharmacol. 858, 172487 10.1016/j.ejphar.2019.172487 31229535

[B24] Rutten-Jacobs L. C. A. Rost N. S. (2020). Emerging insights from the genetics of cerebral small-vessel disease. Ann. N. Y. Acad. Sci. 1471 (1), 5–17. 10.1111/nyas.13998 30618052 PMC6614021

[B25] Shi J. Zhao Y. Wang K. Shi X. Wang Y. Huang H. (2015). Cleavage of GSDMD by inflammatory caspases determines pyroptotic cell death. Nature 526 (7575), 660–665. 10.1038/nature15514 26375003

[B26] Song E. Jahng J. W. Chong L. P. Sung H. K. Han M. Luo C. (2017). Lipocalin-2 induces NLRP3 inflammasome activation via HMGB1 induced TLR4 signaling in heart tissue of mice under pressure overload challenge. Am. J. Transl. Res. 9 (6), 2723–2735. 28670364 PMC5489876

[B27] Szalay G. Martinecz B. Lenart N. Kornyei Z. Orsolits B. Judak L. (2016). Microglia protect against brain injury and their selective elimination dysregulates neuronal network activity after stroke. Nat. Commun. 7, 11499 10.1038/ncomms11499 27139776 PMC4857403

[B28] Vande Walle L. Kanneganti T.-D. Lamkanfi M. (2011). HMGB1 release by inflammasomes. Virulence 2 (2), 162–165. 10.4161/viru.2.2.15480 21422809 PMC3265758

[B29] Wang K. Sun Q. Zhong X. Zeng M. Zeng H. Shi X. (2020). Structural mechanism for GSDMD targeting by autoprocessed Caspases in pyroptosis. Cell 180 (5), 941–955. 10.1016/j.cell.2020.02.002 32109412

[B30] Wang M. Wang F. Peng D. Duan X. Chen W. Xu F. (2020). Tao-Hong Si-Wu decoction alleviates cerebral ischemic damage in rats by improving anti-oxidant and inhibiting apoptosis pathway. Int. J. Pharmacol. 16 (3), 214–222. 10.3923/ijp.2020.214.222

[B31] Wang W. Wang D. Liu H. Sun H. Jiang B. Ru X. (2017). Trend of declining stroke mortality in China: reasons and analysis. Stroke Vasc Neurol. 2 (3), 132–139. 10.1136/svn-2017-000098 28989803 PMC5628381

[B32] Ye J.-X. Wang M. Wang R.-Y. Liu H.-T. Qi Y.-D. Fu J.-H. (2020). Hydroxysafflor yellow A inhibits hypoxia/reoxygenation-induced cardiomyocyte injury via regulating the AMPK/NLRP3 inflammasome pathway. Int. Immunopharm. 82, 106316 10.1016/j.intimp.2020.106316 32088642

[B33] Yin N. Gao Q. Tao W. Chen J. Bi J. Ding F. (2020). Paeoniflorin relieves LPS‐induced inflammatory pain in mice by inhibiting NLRP3 inflammasome activation via transient receptor potential vanilloid 1. J. Leukoc. Biol. 108 (1), 229–241. 10.1002/jlb.3ma0220-355r 32083340

[B34] Yu R. Jiang S. Tao Y. Li P. Yin J. Zhou Q. (2019). Inhibition of HMGB1 improves necrotizing enterocolitis by inhibiting NLRP3 via TLR4 and NF‐κB signaling pathways. J. Cell. Physiol. 234 (8), 13431–13438. 10.1002/jcp.28022 30618088 PMC13483282

[B35] Zhou R. Yazdi A. S. Menu P. Tschopp J. (2011). A role for mitochondria in NLRP3 inflammasome activation. Nature 469 (7329), 221–225. 10.1038/nature09663 21124315

